# Oligomerization of Baculovirus LEF-11 Is Involved in Viral DNA Replication

**DOI:** 10.1371/journal.pone.0144930

**Published:** 2015-12-14

**Authors:** Zhan-Qi Dong, Nan Hu, Jun Zhang, Ting-Ting Chen, Ming-Ya Cao, Hai-Qing Li, Xue-Jiao Lei, Peng Chen, Cheng Lu, Min-Hui Pan

**Affiliations:** 1 State Key Laboratory of Silkworm Genome Biology, Southwest University, Chongqing, 400716, China; 2 Key Laboratory for Sericulture Functional Genomics and Biotechnology of Agricultural Ministry, Southwest University, Chongqing, 400716, China; 3 Institutes of Life Sciences, Chongqing Medical University, Chongqing, 400716, China; Ecole des Mines d'Alès, FRANCE

## Abstract

We have previously reported that baculovirus *Bombyx mori* nucleopolyhedrovirus (BmNPV) late expression factor 11 (*lef-11*) is associated with viral DNA replication and have demonstrated that it potentially interacts with itself; however, whether LEF-11 forms oligomers and the impact of LEF-11 oligomerization on viral function have not been substantiated. In this study, we first demonstrated that LEF-11 is capable of forming oligomers. Additionally, a series of analyses using BmNPV LEF-11 truncation mutants indicated that two distinct domains control LEF-11 oligomerization (aa 42–61 and aa 72–101). LEF-11 truncation constructs were inserted into a *lef-11*-knockout BmNPV bacmid, which was used to demonstrate that truncated LEF-11 lacking either oligomerization domain abrogates viral DNA replication. Finally, site-directed mutagenesis was used to determine that the conserved hydrophobic residues Y58&I59 (representing Y58 and I59), I85 and L88&L89 (representing L88 and L89) are required for LEF-11 oligomerization and viral DNA replication. Collectively, these data indicate that BmNPV LEF-11 oligomerization influences viral DNA replication.

## Introduction

Baculoviruses are a highly diverse group of viruses with large, circular, double-stranded DNA genomes that exclusively infect invertebrates [1,2]. Specific genes have been identified that are required for viral DNA replication, and major advances have been made in understanding the functions of most of these genes in *Alphabaculovirus*; however, the functional roles of DNA replication factors have not been fully elucidated [3–8].

Previous research has identified at least 10 such factors (*lef-1*, *lef-2*, *lef-3*, *lef-7*, *lef-11*, *ie-1*, *ie-2*, *p143*, *dnapol* and *p35*) [3,5–7,9,10]. Late expression factor 11 (*lef-11*) of *Autographa californica* multiple nucleopolyhedrovirus (AcMNPV) encodes a protein with a predicted molecular weight of approximately 13.1 kDa that is required for viral DNA replication and late/very late gene activation[11,12]. Except for the dipteran *Culex nigripalpus* nucleopolyhedrovirus (CuniNPV) genome, *lef-11* is present in all baculovirus genomes. Previous studies have shown that LEF-11 localizes in the nucleus and is essential for viral DNA replication [13]. The nuclear localization signal of *Bombyx mori* nucleopolyhedrovirus (BmNPV) LEF-11 has been identified [14]. Additionally, BmNPV LEF-11 can co-localize with IE-1 in the viral replication center [14]. This evidence suggests that LEF-11 plays an important role in viral DNA replication. In our previous report, we also discovered that LEF-11 might self-interact [14]; however, we do not know the characteristics of LEF-11 oligomerization or its effect on viral proliferation.

In this study, we investigated the roles and characteristics of protein oligomerization with LEF-11 from BmNPV. First, we confirmed that BmNPV LEF-11 forms oligomers using non-reducing sodium dodecyl sulfate-polyacrylamide gel electrophoresis (SDS-PAGE) and a co-immunoprecipitation (IP) assay. Next, a series of truncated proteins were analyzed to reveal the key functional domains involved in oligomerization. To examine the function of LEF-11, we knocked out *lef-11* via λ homologous recombination and identified oligomerization domains that affect virion production and viral DNA replication. We found that residues Y58&I59, I85 and L88&L89 are required for LEF-11 oligomerization and viral DNA replication. Our results suggest that baculovirus LEF-11 oligomerization domains are important for viral DNA replication and viral production.

## Methods

### Cells and Viruses

BmN-SWU1 cells were cultured at 27°C in TC-100 medium (United States Biological, Swampscott, MA, USA) supplemented with 10% (V/V) fetal bovine serum (FBS; PAA Laboratories), penicillin (200 U/mL), and streptomycin (200 U/mL) [15]. *Escherichia coli* strain BW25113 containing the pBAD-gbaA plasmid, the BmNPV genome and the pMON7124 helper plasmid were kindly provided by Ke-Ping Chen (Jiangsu University, China) [16]. Viruses were cultured in BmN-SWU1 cells, and virus titers were determined by 50% tissue culture infectious dose (TCID_50_) assay [16,17].

### Plasmid Construction

To construct plasmids for LEF-11 expression in BmN-SWU1 cells upon transfection, a plasmid vector that contains the *Orgyia pseudotsugata* multi-capsid NPV *OpIE2* early promoter plus a multiple cloning site, pIZ/V5-His (Invitrogen, Carlsbad, CA, USA) was used. The primers used in this study are listed in S1 Table. For LEF-11 oligomerization assessment by western blot, we expressed LEF-11 fused to the cMyc tagged vector pIZ-LEF11^cMYC^. Furthermore, to better understand LEF-11 self-interaction, we constructed the LEF-11 and FLAG tag fusion vectors (pIZ-LEF11^FLAG^) and verified the interaction by co-immunoprecipitation.

To detect the oligomerization domain of LEF-11 in transfected BmN-SWU1 cells, we constructed a series of LEF-11 truncated fragments. However, at only 13.1 kDa, the LEF-11 protein is too small for truncation analysis to detect the corresponding bands. To more clearly detect changes in protein bands, we constructed a series of LEF11-DsRed fusion proteins. First, to identify the oligomerization domain, we divided LEF-11 into its N- and C-termini and subjected these constructs to step-by-step truncation analysis. Eight N-terminal truncation plasmid constructs, including LEF11 (2–61), LEF11 (12–61), LEF11 (22–61), LEF11 (32–61), LEF11 (42–61), LEF11 (52–61), LEF11 (2–51), and LEF11 (2–41), were created. The C-terminal truncation constructs created included LEF11 (62–112), LEF11 (72–112), LEF11 (82–112), LEF-11 (92–112), LEF11 (72–101), LEF11 (72–91) and LEF11 (72–81). Controls included the negative control construct pIZ-DsRed and the full-length fusion construct pIZ-DsRed-LEF11. All constructed plasmids were confirmed by sequencing analysis.

To map the amino acids involved in LEF-11 oligomerization, we used homologous alignment analysis and identified 10 highly conserved hydrophobic amino acids in two oligomerization domains (http://www.ch.embnet.org/software/BOX_form.html). To determine whether oligomerization is dependent on these hydrophobic amino acids, we replaced them with glutamic acid, which is strongly hydrophilic. Site-directed mutations within LEF-11 were synthesized and cloned into a pUC57-simple vector by GenScript (Nanjing, China). The inserts were released using *BamH I* and *Not I* digestion and cloned into pIZ-DsRed or the donor plasmid pFastBacDual-PH-EGFP. The constructs were designated pIZ-DsRed-LEF11-V42E (LEF11-V42E), pIZ-DsRed-LEF11-A44E (LEF11-A44E), pIZ-DsRed-LEF11-F52E (LEF11-F52E), pIZ-DsRed-LEF11-I55E (LEF11-I55E), pIZ-DsRed-LEF11-Y58E&I59E (LEF11-Y58E&I59E), pIZ-DsRed-LEF11-V78E (LEF11-V78E), pIZ-DsRed-LEF11-I85E (LEF11-I85E) and pIZ-DsRed-LEF11-L88E&L89E (LEF11-L88E&L89E).

### Transfections

Plasmid transfections were performed as described previously [17]. Briefly, BmN-SWU1 cells were transfected with 0.8 μg of each plasmid using X-tremeGENE HP DNA Transfection Reagent (Roche). The transfected cells were incubated at 27°C for 6 h, after which the transfection mixture was replaced with fresh TC100.

### Non-Reducing SDS-PAGE Assay

LEF-11 was detected in cell lysates by western blot following 12% SDS-PAGE under reducing and non-reducing conditions. In summary, 80 μL of cell lysate was mixed with 20 μL of 5× SDS-PAGE loading buffer or mixed with 20 μL of non-reducing protein loading buffer (without β-mercaptoethanol) and boiled for 5 min at 95°C. Samples were loaded onto an SDS-PAGE gel. DsRed fusion LEF-11 protein was detected by western blot using anti-DsRed antibody or anti-cMyc antibody (Abcam), as indicated.

### Co-Immunoprecipitation Assays (Co-IP)

Western blot and immunoprecipitation experiments were performed as described previously [16]. BmN-SWU1 cells were transfected with 4 μg of plasmids using 12 μL of X-tremeGENE HP DNA Transfection Reagent (Roche, Risch, Switzerland). The cells were incubated for 72 h at 27°C after the transfection mixture was added and were then washed three times with PBS. BmN-SWU1 cells were resuspended in 900 μL of western and IP lysis buffer (Beyotime, Jiangsu, China) containing 9 μL of phenylmethylsulfonyl fluoride (PMSF). The cell suspensions were lysed at 4°C for 30 min. After the cell lysates were centrifuged at 14,000×g for 30 min at 4°C, 100 μL of each supernatant was collected and subjected to western blotting to analyze the expression of pIZ-LEF11^FLAG^ and pIZ-LEF11^cMYC^ in plasmid-transfected cells. The remaining 800 μL of each supernatant was mixed with 2 μL of anti-FLAG (mouse), anti-cMYC (mouse) or mouse IgG antibody, and each sample was incubated at 4°C overnight. Then, 40 μL of protein A+G agarose beads (Beyotime) was added to the mixture, which was incubated at 4°C for 6 h and then centrifuged at 3,000×g for 5 min at 4°C. The beads were collected by centrifugation and washed three times with western and IP lysis buffer. After 10 μL of 5× SDS loading buffer was added, the beads were boiled in water for 5 min and centrifuged at 13,400×g for 5 min at 4°C. Precipitated proteins were subjected to western blotting as described in a previous report [16]. The antibodies used were rabbit monoclonal anti-FLAG antibody (1:5,000; Sigma, St. Louis, MO, USA) and mouse monoclonal anti-cMYC antibody (1:5,000; Abcam, Cambridge, UK).

### Construction of *lef-11* Deletion and Repair Viruses

The *lef-11* knockout (KO) bacmid was constructed with the λ Red homologous system as previously described [16]. The wild-type (WT) bacmid vBmWT^Bac^ was previously created in our laboratory. To generate *lef-11* KO vBm^lef11KO^, *lef-11* in the bacmid was replaced with a chloramphenicol cassette with the λ Red homologous system. Functional domain deletions and site-directed mutations within LEF-11 were synthesized and cloned into a pUC57-simple vector by GenScript (Nanjing, China). The site-directed mutations within the LEF-11 inserts were released using *BamH I* and *Not I* digestion and cloned into pIZ-DsRed. All functional domain deletions and the site-directed mutations of donor vectors were constructed using *BamH I* and *Not I* digestion, respectively, based on the plasmid pFastBacDual-PH-EGFP. The vectors were confirmed by sequencing, and viral repairs were conducted via Tn7-mediated transposition. The constructs created were bBm^lef11KO-lef11(del42-61)^, bBm^lef11KO-lef11(del72-91)^, bBm^lef11KO-lef11(del42-61/72-91)^, bBm^lef11KO-V42E^, bBm^lef11KO-A44E^, bBm^lef11KO-F52E^, bBm^lef11KO-I55E^, bBm^lef11KO-Y58E&I59E^, bBm^lef11KO-V78E^, bBm^lef11KO-I85E^ and bBm^lef11KO-L88E&L89E^. Successful transposition was confirmed by PCR.

### BV Production in BmN-SWU1 Cells

BmN-SWU1 cells were transfected with 4 μg of recombinant BmNPV bacmid. Budded viruses in the culture medium were separated from infected cells by centrifugation at different time points. Each single-step growth curve experiment was replicated three times. The virus titers of all samples were determined and expressed as the TCID_50_ in BmN-SWU1 cells.

### Quantitative Real-Time PCR (qRT-PCR) DNA Replication Assay

To analyze viral DNA replication, a quantitative PCR (qPCR) assay was performed as previously described [16]. To prepare viral DNA for analysis, BmN-SWU1 cells (1×10^6^ cells) were transfected with 2 μg of bacmid DNA (bBm^WTBac^, bBm^lef11KO^, bBm^lef11KO-LEF11:cMYC^ and bBm^lef11KO-LEF11M:cMYC^). Total DNA was extracted from each sample using a Classic Genomic DNA Isolation Kit (Promega, Madison, WI, USA) following the manufacturer’s instructions. Samples were analyzed using the CFX96TM Real-Time System (Bio-Rad, California, USA) under the following conditions: 95°C for 40 s followed by 40 cycles at 95°C for 5 s and 60°C for 15 s, with 500 μM of each primer.

## Results

### LEF-11 Exists in an Oligomeric Form

**Fig 1:**
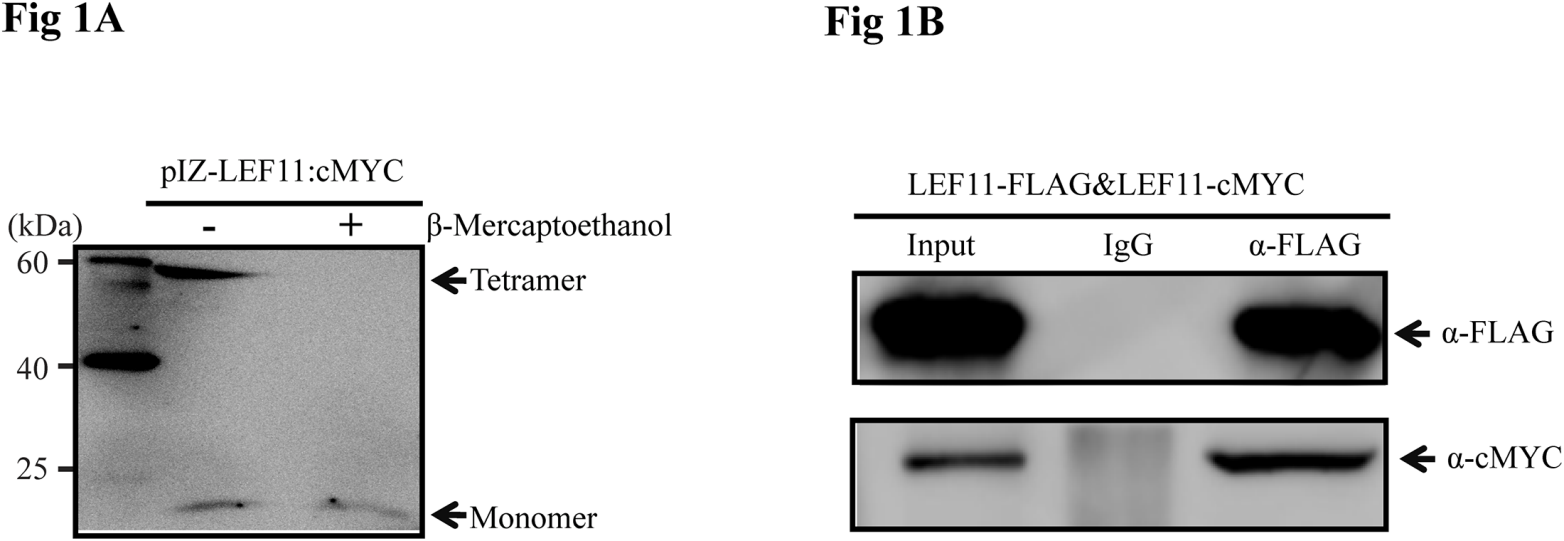
LEF-11 exists in an oligomeric form. **(A) Oligomerization state of LEF-11, as determined by non-reducing SDS-PAGE analysis**. BmN-SWU1 cells were transfected with pIZ-LEF-11^cMYC^ plasmid. The cells were collected and lysed at 72 hours post transfection (h.p.t.). The samples were mixed with non-reducing protein loading buffer (without β-mercaptoethanol) without boiling, and α-cMYC antibody was added to detect oligomerization. +: with β-mercaptoethanol; -: without β-mercaptoethanol. **(B) Co-immunoprecipitation (Co-IP) assays to verify LEF-11 homo-oligomerization.** BmN-SWU1 cells were co-transfected with LEF11-FLAG and LEF11-cMYC plasmids. At 72 h.p.t., cells were collected and lysed for immunoprecipitation (IP) with α-FLAG antibody. Western blots were probed with α-FLAG antibody to detect the expression and immunoprecipitation of the input proteins and with α-cMYC antibody to detect co-immunoprecipitated LEF11-cMYC. Input, cell lysates; mouse IgG, IP with control mouse IgG.

To define the structure of BmNPV LEF-11, we used non-reducing SDS-PAGE analysis and found that this protein exists mainly as an oligomer; however, LEF-11 could be detected only as a monomer after treatment with β-mercaptoethanol (Fig 1A). We speculate that the protein band of approximately 60 kDa represents a LEF-11 tetramer. These results indicate that the active form of LEF-11 is primarily oligomeric. Further analysis by Co-IP verified that LEF-11 interacts with itself, as the LEF-11:cMYC fusion protein co-precipitated with the LEF-11:FLAG fusion protein in co-transfected BmN-SWU1 cells (Fig 1B). These results further strengthened our conclusions that LEF-11 interacts with itself and mainly exists in tetrameric form.

### LEF-11 Possesses Two Oligomerization Domains

**Fig 2:**
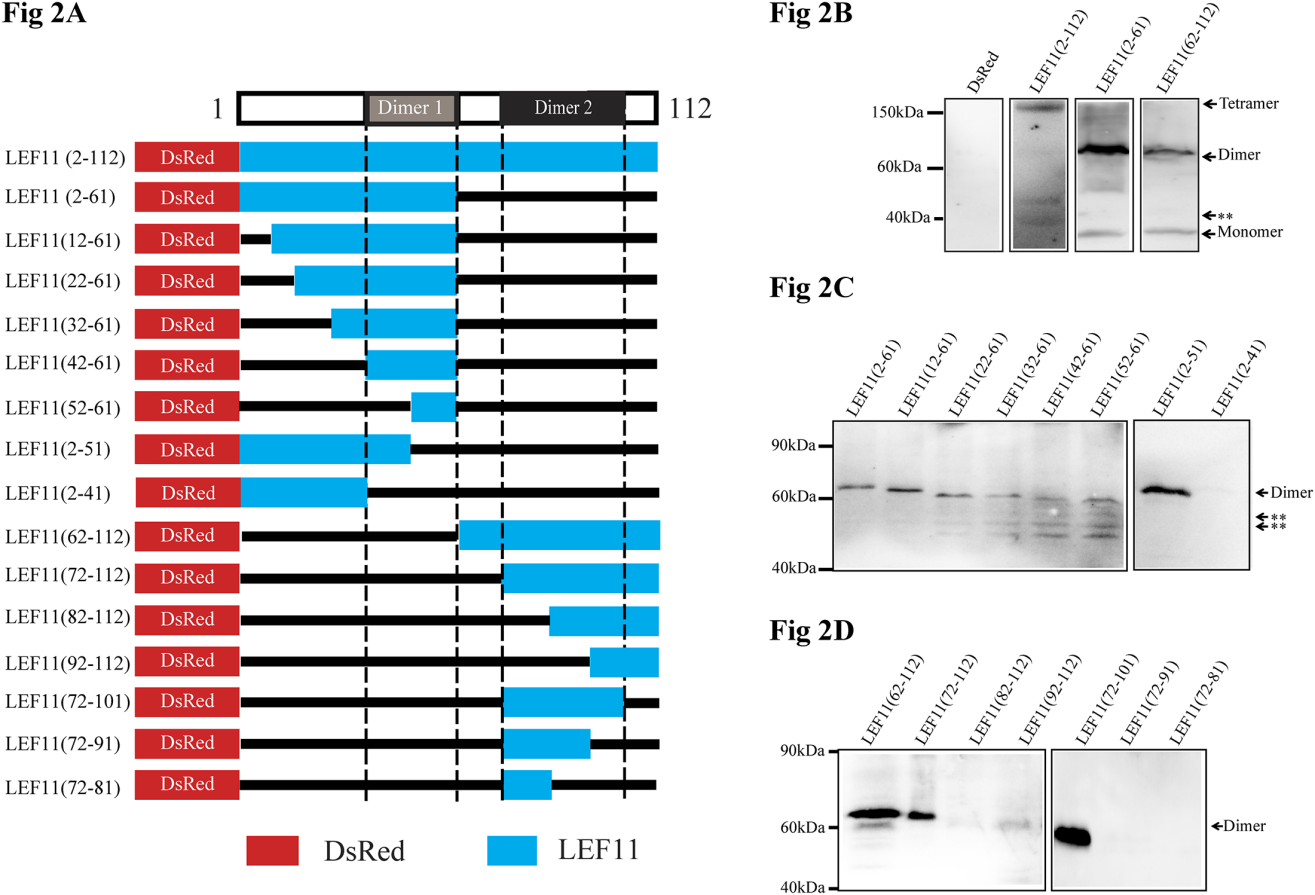
Truncation mutants were used to identify oligomerization domains. **(A) Schematic diagram of LEF-11 truncation mutants.** The blue-shaded boxes represent the regions of LEF11 (as indicted by the amino acid numbers on the left). Each protein was fused at the N-terminus with the same DsRed region (DsRed, red box). Straight lines represent deleted amino acids. **(B-D) Oligomerization of LEF-11 truncation mutants.** BmN-SWU1 cells were transfected with pIZ-DsRed-LEF-11 plasmids for non-reducing SDS-PAGE analysis of LEF-11 truncation mutants. **B. LEF-11 N-terminal and -C-terminal halves. C. N-terminal half truncations. D. C-terminal half truncations.** Asterisks represent non-specific bands.

To further study the characteristics of LEF-11 oligomerization, BmN-SWU1 cells were transfected with the plasmid pIZ-DsRed-LEF11 or with pIZ-DsRed-LEF11 truncation mutants and the expressed products analyzed by western using non-reducing SDS-PAGE (Fig 2A). We detected dimerization of DsRed-LEF11 (2–61) and DsRed-LEF11 (62–112) products because similar bands of approximately 70 kDa could be observed for both (Fig 2B). These results indicate that LEF-11 may possess more than one dimer domain. We first analyzed a series of truncation mutants of DsRed-LEF11 (2–61). The results showed that the dimer bands of LEF11 (2–61), LEF11 (12–61), LEF11 (22–61), LEF11 (32–61), LEF11 (42–61) and LEF11 (52–61) could be detected, with the dimer bands gradually becoming weaker as LEF-11 was further truncated. This results shows that LEF11 (52–61), the N-terminal half of LEF-11, is important for dimer formation. To further analyze the dimer formation characteristics of the N-terminal half of LEF-11, we gradually truncated LEF-11 from the opposite direction. The results showed that LEF11 (2–51) forms a dimer and that LEF11 (2–41) does not form a dimer. These results suggest that LEF11 (42–51) of LEF-11 is also very important for dimer formation. Thus, LEF11 (42–61) is the essential functional domain, whereas LEF11 (2–41) has a role in maintaining a stable dimerization structure (Fig 2C). We conducted the same analysis for DsRed-LEF11 (62–112). Non-reducing SDS-PAGE revealed that LEF11 (62–112) and LEF11 (72–112) were capable of stably dimerizing, unlike LEF11 (82–112) and LEF11 (92–112). This result indicates that LEF11 (72–81) is necessary for dimerization. We used the same method to analysis the truncated C-terminal half of LEF-11 from the opposite direction. The results show that LEF11 (72–101) can form dimers, whereas LEF11 (72–91) cannot (Fig 2D). All these results indicate that LEF11 (72–101) is essential for LEF-11 dimerization. The above results also show that LEF-11 has two domains: domain 42–61, located in the N-terminal region of LEF-11, and domain 72–101, located in the C-terminal region of LEF-11. When LEF-11 is fragmented into N and C terminal fragments, domain 42–61 promotes the dimerization of the N-terminal half and domain 72–101 promotes the dimerization of the C-terminal half. This finding led to the hypothesis that the existence of LEF-11 in the form of a tetramer is due to the concerted action of both domains through the interaction of four LEF-11 monomers.

### Oligomerization Domains Are Essential for Viral DNA Replication

**Fig 3:**
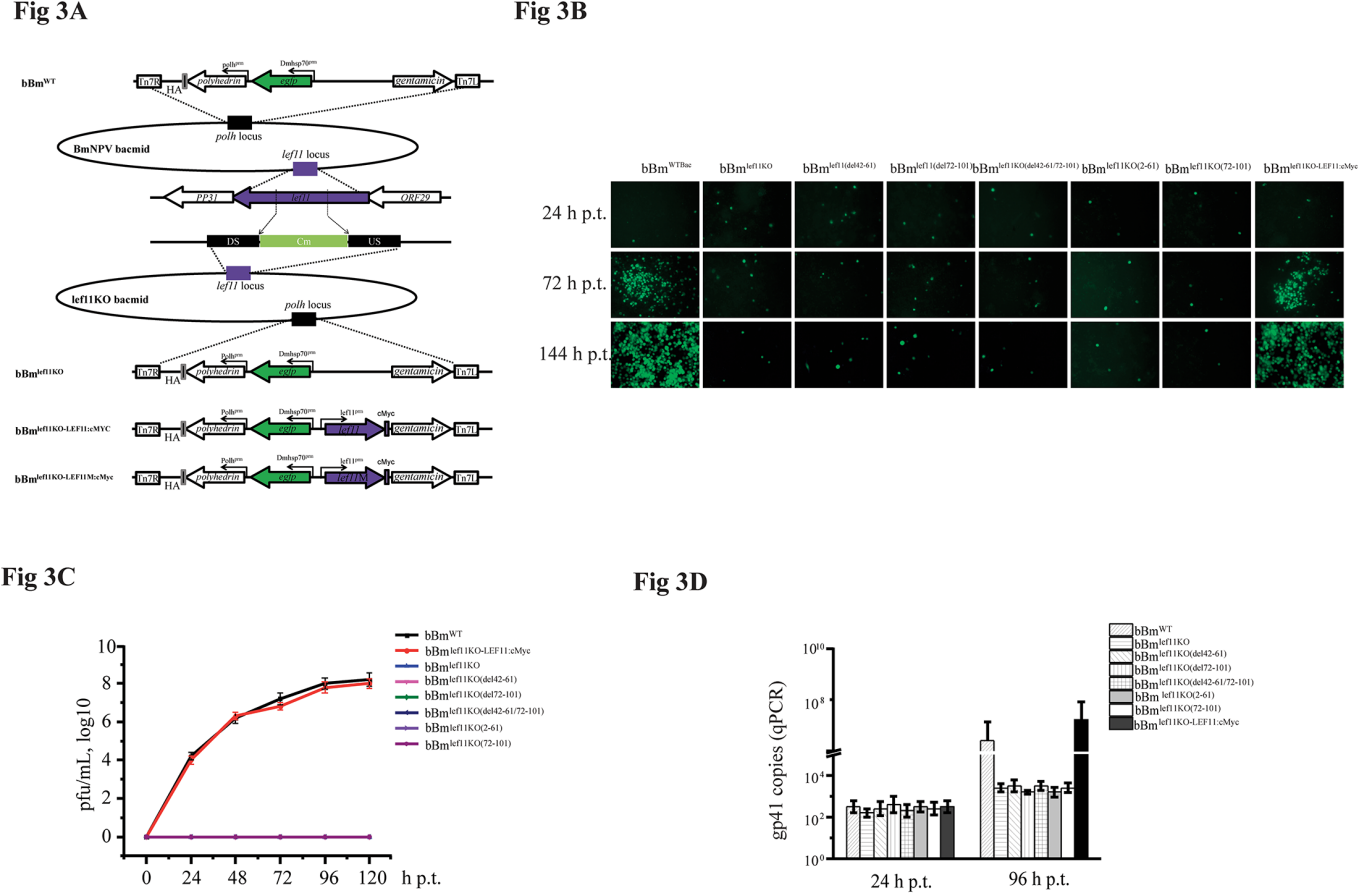
Oligomerization domains are essential for virus DNA replication. **(A) Schematic diagram of the bacmids bBm** ^**WT**^ **, bBm** ^**lef11KO**^ **, bBm** ^**lef11KO-LEF11:cMYC**^ **and bBm** ^**lef11KO-LEF11M:cMYC**^. The majority of *lef-11* was deleted via replacement with a *cm* gene resistance cassette via homologous recombination in *E*. *coli*, generating lef11-KO. The lower part of the figure shows the mutant genes inserted into the polyhedrin locus of lef11-KO by Tn7-mediated transposition for each of the repair viruses generated. The mutant virus was repaired via transfer vectors encoding polyhedrin, EGFP and various mutants to generate bBm^lef11KO^, bBm^lef11KO-LEF11:cMYC^ and bBm^lef11KO-LEF11M:cMYC^. The expression of *lef-11* was driven by its own promoters, and *lef-11* mutants were tagged with sequences encoding the cMYC epitope tag at the 3’ end of the open reading frame (ORF). **(B) Fluorescence-based analysis of virus replication in cells transfected with LEF-11 in bBm** ^**WT**^ **, bBm** ^**lef11KO**^ **, bBm** ^**lef11KO-LEF11:cMYC**^ **and bBm** ^**lef11KO-LEF11M:cMYC**^. BmN-SWU1 cells were transfected with each bacmid and observed under a fluorescence microscope at 24, 72 and 144 h.p.t. **(C) Analysis of LEF-11 BV titers in bBm** ^**WT**^ **, bBm** ^**lef11KO**^ **, bBm** ^**lef11KO-LEF11:cMYC**^ **and bBm** ^**lef11KO-LEF11M:cMYC**^. Titration of BV production at 96 h.p.t. from BmN-SWU1 cells transfected with the indicated bacmids. Titers were determined by end-point dilution assays. Error bars indicate standard error. **(D) DNA replication of bBm** ^**WT**^ **, bBm** ^**lef11KO**^ **, bBm** ^**lef11KO-LEF11:cMYC**^ **and bBm** ^**lef11KO-LEF11M:cMYC**^ **in transfected BmN-SWU1 cells.** BmN-SWU1 cells were transfected with DNA from all indicated bacmids. At regular intervals post transfection, total DNA was extracted, and viral DNA was quantified by qPCR. Each time point represents the average of two independent replicates. bBm^lef11KO-LEF11M:cMYC^ represents bBm^lef11KO-lef11(del42-61)^, bBm^lef11KO-lef11(del42-61)^ and bBm^lef11KO-lef11(del42-61/72-101)^.

To determine the role of the LEF-11 oligomerization domain during viral infection in BmN-SWU1 cells, we constructed a *lef-11* mutant bacmid (Fig 3A). Following bacmid transfection, microscopy indicated the presence of reporter enhanced green fluorescent protein (EGFP) fluorescence. At 24 hours post-transfection (h.p.t.), the same numbers of green fluorescent cells were observed in samples transfected with bBm^WT^, bBm^lef11KO^, bBm^lef11KO-LEF11:cMYC^ and bBm^lef11KO-LEF11M:cMYC^. At 72 h.p.t., EGFP fluorescence had spread from the transfected cells to the surrounding cells after treatment with the bBm^WT^ and bBm^lef11KO-LEF11:cMYC^ bacmids; however, the amount of fluorescence did not increase after treatment with the bBm^lef11KO^ and bBm^lef11KO-LEF11M:cMYC^ bacmids until 144 h.p.t., indicating that the virus did not spread from the transfected cells treated with the *lef-11* KO and dimerization-domain KO bacmids (Fig 3B).

Virus titers were used to evaluate the effects of LEF-11 mutants on viral production. Virus titers of bBm^WT^ and bBm^lef11KO-LEF11:cMYC^ steadily increased following transfection, whereas the virus titers of bBm^lef11KO^ and bBm^lef11KO-LEF11:cMYC^ did not increase at any detectable rate after 120 h.p.t. (Fig 3C). These data suggest that bacmids encoding dimerization domain KO proteins are incapable of producing infectious viruses. At 24 h.p.t., similar signals from viral DNA could be detected in transfected cells, indicating that the equivalent amount of DNA was present. Analysis of viral DNA replication from 24 to 96 h.p.t. revealed that a large amount of viral DNA was generated by the bBm^WT^ and bBm^lef11KO-LEF11:cMYC^ bacmids; however, viral DNA production did not increase with bBm^lef11KO^ and bBm^lef11KO-LEF11M:cMYC^ bacmid treatments. These data indicate that the bBm^WT^ and bBm^lef11KO-LEF11:cMYC^ bacmids can initiate a secondary infection, resulting in an increase in the number of cells containing viral DNA, whereas the bBm^lef11KO^ and bBm^lef11KO-LEF11:cMYC^ bacmids cannot replication in transfected cells (Fig 3D). qPCR results indicate that deletion of the *lef-11* oligomerization domain affects viral DNA replication in BmN-SWU1 cells.

### Hydrophobic Amino Acids Are Required for LEF-11 Oligomerization in Transfected Cells

**Fig 4:**
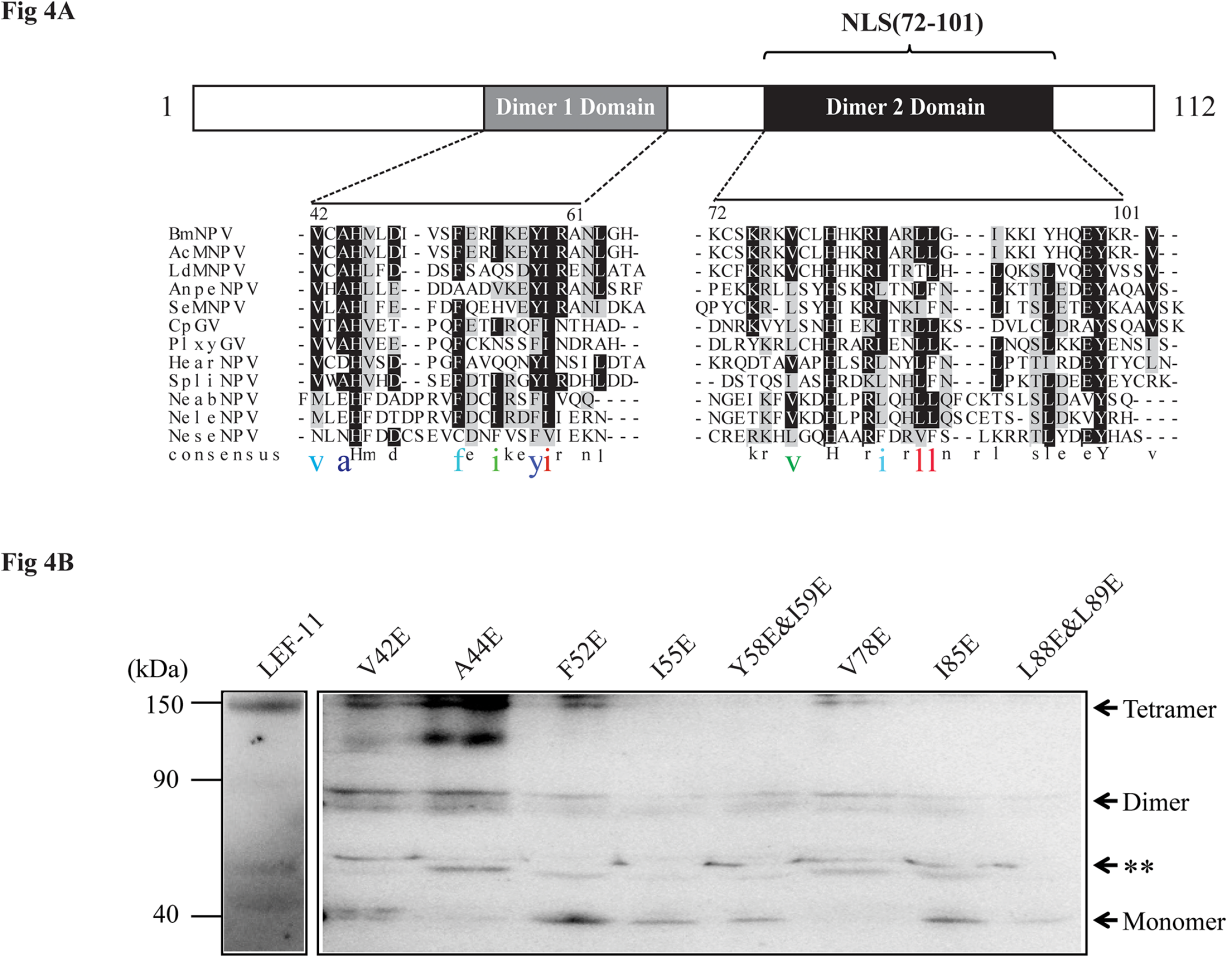
Mutagenesis studies of the oligomerization domain. **(A) Amino acid sequence similarity between baculovirus LEF-11 mutants.** The putative BmNPV LEF-11 oligomerization domains were aligned with other baculovirus species using ClustalW. The core oligomerization domains are shown in boxes. Identical and similar residues are highlighted in black and gray, respectively, using the Boxshade Server (http://www.ch.embnet.org/software/BOX_form.html). The mutated residues in the oligomerization domain are shown in color and large type. **(B) BmN-SWU1 cells were transfected with LEF-11 site-directed mutant plasmids and analyzed via non-reducing SDS-PAGE analysis.** At 72 h.p.t., cells were collected and lysed. The samples were mixed with non-reducing protein loading buffer (without β-mercaptoethanol) without boiling and were mixed with α-DsRed antibody to detect oligomerization. Asterisks represent non-specific bands.

Previous studies have shown that the general stability of the oligomers is maintained by hydrophobic amino acids. To demonstrate the characteristics of LEF-11 oligomers, the amino acid sequences of two oligomerization domains were compared via homology analysis. The results show that hydrophobic amino acid structures are highly conserved (Fig 4A). To analyze whether these hydrophobic amino acids can determine oligomerization, we conducted site-directed mutagenesis against the highly hydrophilic glutamic acids at certain sites, including V42E, A44E, F52E, I55E, Y58E&I59E, V78E, I85E and L88E&L89E (Fig 4A). All site-directed mutagenesis products were analyzed by non-reducing SDS-PAGE. Mutants A44E and V78E were only detected in oligomeric forms, whereas mutants V42E and F52E were detected in both oligomeric and monomeric forms (Fig 4B). I55E, YE58&I59E, I85E and L88E&L89E were detected only in dimeric and monomeric forms (Fig 4B). These results demonstrate that hydrophobic amino acids affect oligomerization.

### Hydrophobic Amino Acids in the LEF-11 Oligomerization Domain Are Required for Viral DNA Replication

**Fig 5:**
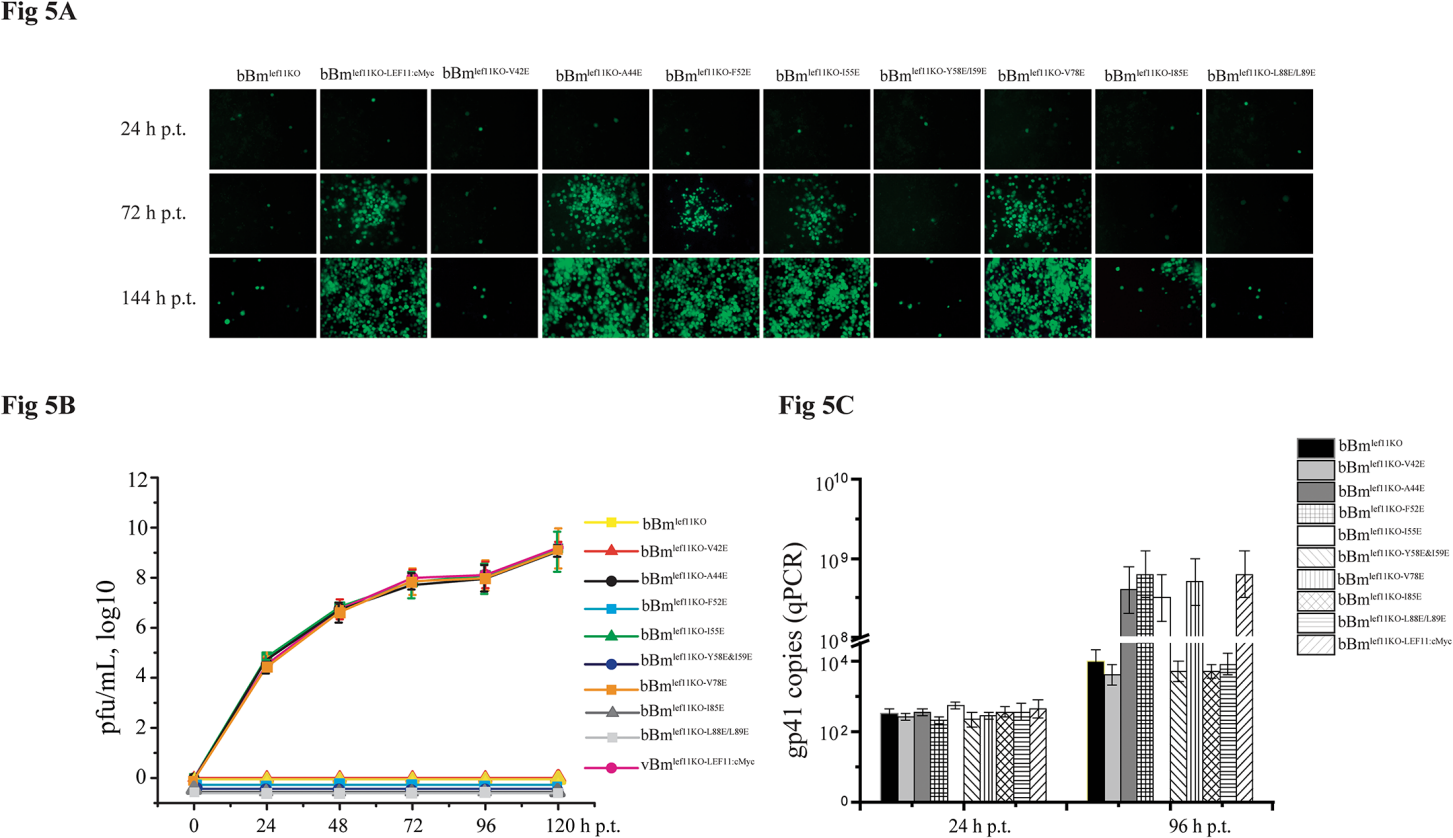
Hydrophobic amino acids in the LEF-11 oligomerization domain are required for viral DNA replication. **(A) Fluorescence-based analysis of LEF-11 in bBm** ^**lef11KO**^ **, bBm** ^**lef11KO-LEF11:cMYC**^ **and bBm** ^**lef11KO-LEF11M:cMYC**^ **(V42E, A44E, F52E, Y58E &I59E, V78E, I85E and L88E&L89E).** BmN-SWU1 cells were transfected with each bacmid and observed under a fluorescence microscope at 24, 72 and 144 h.p.t. **(B) Analysis of LEF-11 BV titers in bBm** ^**lef11KO**^ **, bBm** ^**lef11KO-LEF11:cMYC**^ **and bBm** ^**lef11KO-LEF11M:cMYC**^. Titration of BV production in BmN-SWU1 cells transfected with the indicated bacmids. Titers were determined by end-point dilution assays. Error bars indicate standard error. **(C) Replication of bBm** ^**lef11KO**^ **, bBm** ^**lef11KO-LEF11:cMYC**^ **and bBm** ^**lef11KO-LEF11M:cMYC**^ **in transfected BmN-SWU1 cells.** BmN-SWU1 cells were transfected with DNA from all indicated bacmids. At various h.p.t., total DNA was extracted, and viral DNA was quantified by qPCR. Each time point represents the average of two independent replicates.

To determine the sequences of the BmNPV LEF-11 hydrophobic amino acids necessary for DNA replication, 10 hydrophobic amino acids from oligomerization domains were replaced one at a time by glutamic acid, and the DNA encoding the glutamic-substituted *lef-11* was reintroduced into bBm^lef11-KO^, creating bBm^lef11KO-LEF11M:cMYC^. The amount of green fluorescence from all transfection bacmids was consistent at 24 h.p.t.; these results showed that these bacmids had the same transfection efficiency (Fig 5A). By 72 h.p.t., green fluorescence from transfected cells had spread to neighboring cells from the A44E, F52E, I55E and V78E mutant bacmids. No spread was observed from the green fluorescent cells of the V42E, Y58E&I59E, I85E, and L88E&L89E mutant bacmids by 144 h.p.t. Interestingly, the virus mutant I85 exhibited a limited spread of fluorescence at 144 h.p.t. (Fig 5A). These results suggest that individual hydrophobic amino acids in the oligomerization domains of LEF-11 are important for viral DNA replication.

Virus titers steadily increased in the transfections with A44E, F52E, I55E and V78E mutation bacmids following transfection, whereas the virus titers for the V42E, Y58E&I59E, I85E and L88E&L89E mutations had not increased by 120 h.p.t. (Fig 5B). These data indicate that the V42E, Y58E&I59E, I85E and L88E&L89E mutants cannot produce infectious viruses and that A44E, F52E, I55E and V78E mutants do not affect BV production. Equivalent replication from each bacmid was detected in transfected cells at 24 h.p.t., indicating that all bacmid have equal amounts of DNA present. Viral DNA replication from the A44E, F52E, I55E and V78E amino acid substitution bacmids steadily increased from 24 to 96 h.p.t.; however, the levels of viral DNA replication observed for the V42E, Y58E&I59E, I85E and L88E&L89E mutant bacmids did not increase. These data suggest that the V42E, Y58E&I59E, I85E and L88E&L89E mutants cannot replicate in transfected cells (Fig 5C). qPCR results indicate that mutations in the hydrophobic amino acids V42E, Y58&I59E, I85E and L88E&L89E affect viral DNA replication in BmN-SWU1 cells.

## Discussion

LEF-11 is necessary for viral DNA replication during the infection cycle, and our previous studies have shown that LEF-11 may exist as homo-dimers during viral replication [11,12,14]. Although some characteristics of the LEF-11 protein were understood, the oligomerization of baculovirus LEF-11 and its influence on virus replication are not yet well defined [14]. In this study, we generated a series of truncated LEF-11 proteins and tested their oligomerization profiles.

Homo-oligomers are often the active forms of many DNA replication-specific expression factors in baculovirus [18,19]. Previous studies have shown that oligomers of several genes, including *lef-1*, *lef-2*, *ie-1*, *ie-2*, *lef-5*, *dbp*, *ha44* and *cg30*, are required for regulating DNA replication during AcMNPV proliferation [20–26]. In addition, the AcMNPV gene *lef-3* can interact with itself to form a homo-oligomer, indicating that the entire LEF-3 may also be essential to viral DNA replication [19,27]. Similarly, our results demonstrate that tetramers are formed by the BmNPV LEF-11 protein and that replication of the DNA of most mutants lacking the tetramer was blocked. We previously demonstrated that LEF-11 co-localizes with IE-1 in the nucleus and interacts with LEF-3 [14]. Both observations support the hypothesis that LEF-11 may form a complex with the viral DNA replication machinery, thus playing an important role in the regulation of viral replication.

In our study, 4 strains of mutated virus completely lacking the LEF-11 tetramer were generated, and the DNA replication of the mutated strains, except for the I55-mutant strain, was blocked (Fig 4B). The expression levels of the tetramers of these mutant stains were compared by western blot analysis. The result shows that the tetramer level of V78E, one strain with incomplete tetramer removal, was significantly lower than were those of the other 2 strains with tetramers (A44E and F52E). However, no significant difference in virus replication was detected between these strains, suggesting that even a low level of tetramer formation is sufficient for virus DNA replication. Therefore, several tetramer molecules may exist, but under detectable levels, in the host cells of the I55E mutant. Additionally, a small quantity of tetramers can activate the replication of I55E. Another possible reason may be the unstable structure of the tetramer in I55E, which causes it to be undetectable. In addition, many previous studies have reported that the formation of oligomers is an extremely unstable process [28–31]. However, principally, we considered that oligomerization assays were performed with cells transfected with plasmids expressing LEF-11 mutants alone, without any other viral proteins. In contrast, LEF-11 is expressed from bacmids in the context of viral infection. Therefore, we cannot detect the formation of tetramers of the I55E mutant at the protein level, although this mutant remained able to generate infectious BVs. However, the reason why no LEF-11 tetramer was detected by western blot analysis requires further exploration; this analysis is part of our future research plan. Previous studies have indicated that LEF-11 has a conserved zinc finger domain; thus, the V42E mutant may affect the zinc finger functionality. In addition, LEF-11 must have functional domains that are unknown at present. Therefore, the V42E mutation may affect the function of the *lef-11* gene itself but not its tetramer.

Interestingly, we discovered that the sequence of the nuclear localization signal overlapped with dimer domain 72–101 of LEF-11. Unstable oligomers may be stabilized through interaction with other viral proteins in the replisome once these oligomers enter the nucleus during viral infection. This interaction is probably the reason why mutations in domain 72–101 that affects oligomerization correlate with a lack of virus replication and why not all mutations in domain 42–61 that affect oligomerization imply replication deficiency. Future research focusing on the relationship between oligomerization and nuclear import may more clearly explain the current results.

Oligomerization domains are essential for proteins to form oligomers. In our study, we defined two highly conserved oligomerization domains in LEF-11, which are located between amino acids 42–61 and 72–101 (Fig 4A). Previously, Lin et.al demonstrated that the LEF-11 protein contains a single putative zinc finger motif in its N-terminal region and a basic charged region in its C-terminal region [12]. Moreover, Zhang et.al identified a nuclear location signal (amino acids 72–101) in the C-terminal region of LEF-11 and demonstrated that baculovirus LEF-11 co-localizes with IE-1 at viral DNA replication sites [14]. Considering these findings, a model of how LEF-11 functions similar to IE1 was generated as follows [6,29]. After the LEF-11 protein is synthesized in the cytoplasm, it can form dimers/tetramers in the cytoplasm and then enter the host nucleus by binding directly to the host importin ɑ-3 protein. Then, LEF-11 anchors to the virogenic stroma and can regulate viral replication by a mechanism that is not yet fully understood. Although this model only vaguely explains the involvement of LEF-11 in virus replication, the current study provides novel insight for future studies of the molecular mechanism of baculovirus replication.

In summary, we have identified two oligomerization domains of LEF-11 and determined the mechanism regulating viral DNA replication. The hydrophobic amino acids A42E, Y58E&I59E, I85E and L88E&L89E are critical for viral DNA replication; Y58&I59, I85 and L88&L89 are also required for LEF-11 oligomerization, suggesting that oligomerization is a prerequisite for LEF-11 function in DNA replication. Although further work is required to determine how LEF-11 oligomerization contributes to viral DNA replication and the molecular mechanism of LEF-11 regulation of virus replication, the work presented here, combined with the function of the key factor of baculovirus replication, can further reveal the mechanism of baculovirus replication.

## Supporting Information

S1 Table All the primers used in this study. (TIF)
