# Supplementary figures and images for: Oligomerization of Baculovirus LEF-11 Is Involved in Viral DNA Replication
Source: PLoS One. 2015 Dec 14;10(12):e0144930. doi: 10.1371/journal.pone.0144930 (PMC4678028; doi:10.1371/journal.pone.0144930)

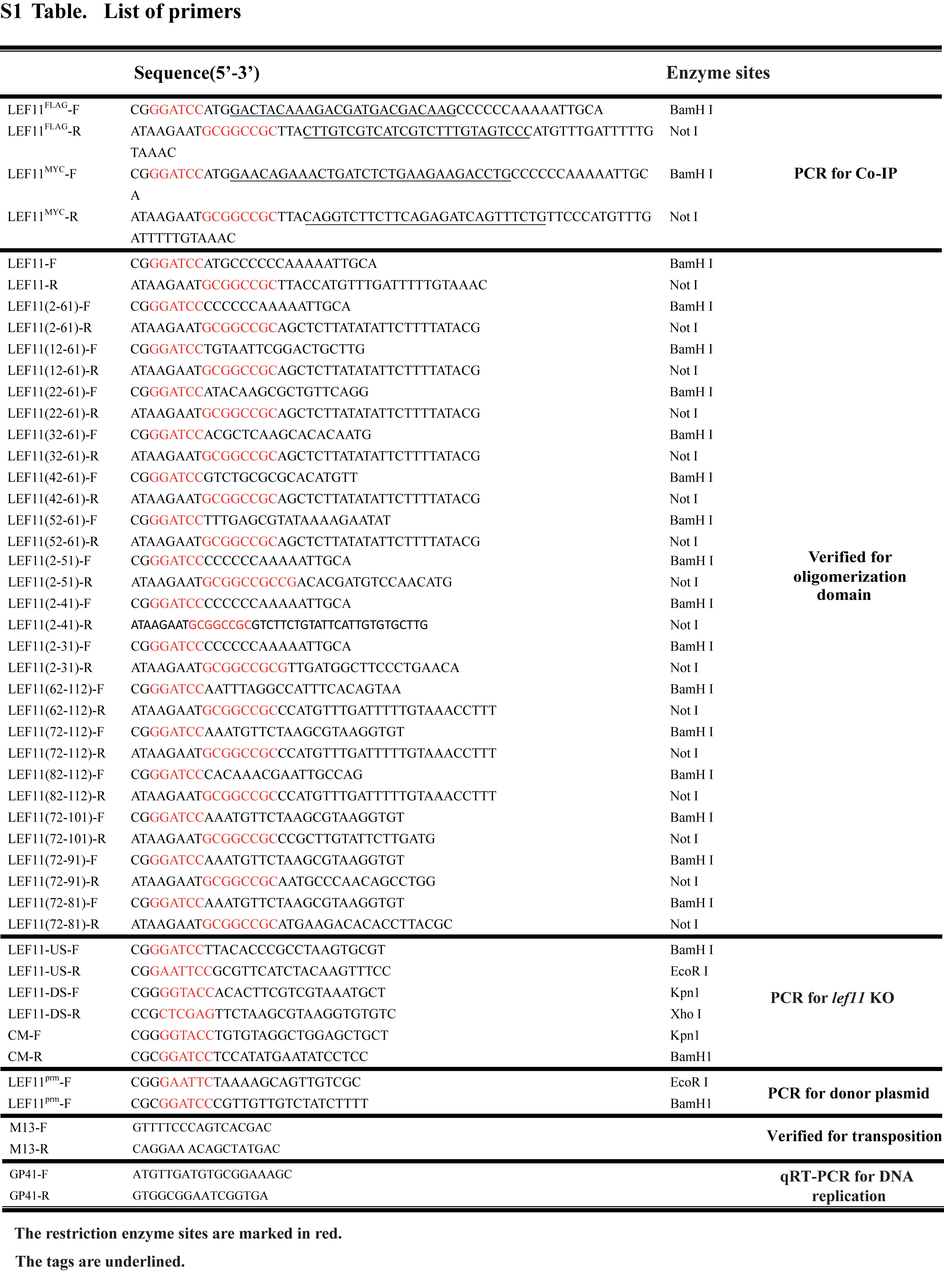

Supplement: S1 Table — (TIF) [file pone.0144930.s001.tif]
